# Reliability and Validity of Noncognitive Ecological Momentary Assessment Survey Response Times as an Indicator of Cognitive Processing Speed in People’s Natural Environment: Intensive Longitudinal Study

**DOI:** 10.2196/45203

**Published:** 2023-05-30

**Authors:** Raymond Hernandez, Claire Hoogendoorn, Jeffrey S Gonzalez, Haomiao Jin, Elizabeth A Pyatak, Donna Spruijt-Metz, Doerte U Junghaenel, Pey-Jiuan Lee, Stefan Schneider

**Affiliations:** 1 Center of Economic and Social Research University of Southern California Los Angeles, CA United States; 2 Ferkauf Graduate School of Psychology Yeshiva University Bronx, NY United States; 3 Fleischer Institute for Diabetes and Metabolism, Division of Endocrinology Department of Medicine, Albert Einstein College of Medicine Bronx, NY United States; 4 School of Health Sciences, Faculty of Health and Medical Sciences University of Surrey Guildford United Kingdom; 5 Chan Division of Occupational Science and Occupational Therapy University of Southern California Los Angeles, CA United States; 6 Keck School of Medicine University of Southern California Los Angeles, CA United States; 7 Department of Psychology University of Southern California Los Angeles, CA United States

**Keywords:** cognitive performance, processing speed, ecological momentary assessment, ambulatory assessment, type 1 diabetes, survey response times, paradata, chronic illness, smartphone, mobile health, mHealth, mobile phone

## Abstract

**Background:**

Various populations with chronic conditions are at risk for decreased cognitive performance, making assessment of their cognition important. Formal mobile cognitive assessments measure cognitive performance with greater ecological validity than traditional laboratory-based testing but add to participant task demands. Given that responding to a survey is considered a cognitively demanding task itself, information that is passively collected as a by-product of ecological momentary assessment (EMA) may be a means through which people’s cognitive performance in their natural environment can be estimated when formal ambulatory cognitive assessment is not feasible. We specifically examined whether the item response times (RTs) to EMA questions (eg, mood) can serve as approximations of cognitive processing speed.

**Objective:**

This study aims to investigate whether the RTs from noncognitive EMA surveys can serve as approximate indicators of between-person (BP) differences and momentary within-person (WP) variability in cognitive processing speed.

**Methods:**

Data from a 2-week EMA study investigating the relationships among glucose, emotion, and functioning in adults with type 1 diabetes were analyzed. Validated mobile cognitive tests assessing processing speed (Symbol Search task) and sustained attention (Go-No Go task) were administered together with noncognitive EMA surveys 5 to 6 times per day via smartphones. Multilevel modeling was used to examine the reliability of EMA RTs, their convergent validity with the Symbol Search task, and their divergent validity with the Go-No Go task. Other tests of the validity of EMA RTs included the examination of their associations with age, depression, fatigue, and the time of day.

**Results:**

Overall, in BP analyses, evidence was found supporting the reliability and convergent validity of EMA question RTs from even a single repeatedly administered EMA item as a measure of average processing speed. BP correlations between the Symbol Search task and EMA RTs ranged from 0.43 to 0.58 (*P*<.001). EMA RTs had significant BP associations with age (*P*<.001), as expected, but not with depression (*P=.*20) or average fatigue (*P=.*18). In WP analyses, the RTs to 16 slider items and all 22 EMA items (including the 16 slider items) had acceptable (>0.70) WP reliability. After correcting for unreliability in multilevel models, EMA RTs from most combinations of items showed moderate WP correlations with the Symbol Search task (ranged from 0.29 to 0.58; *P*<.001) and demonstrated theoretically expected relationships with momentary fatigue and the time of day. The associations between EMA RTs and the Symbol Search task were greater than those between EMA RTs and the Go-No Go task at both the BP and WP levels, providing evidence of divergent validity.

**Conclusions:**

Assessing the RTs to EMA items (eg, mood) may be a method of approximating people’s average levels of and momentary fluctuations in processing speed without adding tasks beyond the survey questions.

## Introduction

### Background

Various illnesses are risk factors for decreased cognitive performance, including type 1 diabetes (T1D), type 2 diabetes, depression, and cardiovascular disease [1-4], making the assessment of cognition in these populations important. Although the measurement of the cognitive performance of individuals with various chronic illnesses in their real-world environments is potentially useful (eg, as it provides ecologically valid measures) [5], formal ambulatory cognitive assessment is at times infeasible because of limited resources and time demands already placed on participants from other ambulatory assessment tasks. We attempt to capitalize on the growing use of the ecological momentary assessment (EMA) methodology in behavioral health research [6-8] and propose a novel approach for assessing a central cognitive measure, processing speed, using EMA survey paradata. Paradata are data about the response process, such as response times (RTs) when answering survey questions [9,10], that can be passively captured alongside survey responses. Measuring cognitive performance via EMA paradata could create novel opportunities for researchers to examine respondents’ real-time cognitive performance in studies in which formal ambulatory cognitive testing cannot be readily implemented. This, in turn, could allow for a more frequent investigation of the antecedents, correlates, and consequences of changes in processing speed across a wider range of individuals and populations with chronic conditions.

Several behavioral health-focused EMA studies have used formal ambulatory cognitive tests in various populations, including individuals with T1D [11-13], breast cancer survivors [14], and people with fibromyalgia [15]. Formal ambulatory cognitive assessments have been viewed as a gold standard for capturing people’s cognitive performance in their natural environment and overcome several limitations of traditional cognitive testing, including the ability to represent cognitive performance in real-world settings, increased frequency with which tests can be administered, and the ability to capture changes over short time frames [5]. However, formal ambulatory cognitive assessments often require more time to complete than other EMA measures. For instance, assessing a single aspect of cognition often requires 45 to 60 seconds [5,16], whereas the measurement of constructs such as stress requires the completion of a single item. Therefore, to administer formal ambulatory cognitive tests, researchers must at times limit the number of survey items included to keep the overall time to complete EMA surveys manageable. They also often require a costly setup and the use of specific programs or apps, which can be obstacles to implementation for many researchers. The difficulties in implementing formal ambulatory assessments limit our ability to more frequently investigate cognitive performance in everyday real-world settings in populations with chronic conditions. When considering T1D specifically, ambulatory cognitive performance has rarely been assessed [12,13,16], limiting our understanding of time-varying correlates and ultimately our understanding of the multifactorial pathways connecting diabetes to cognitive performance and decline.

Cognitive performance may potentially be inferred from EMA survey paradata (eg, RTs to mood items) and thus could be a means to approximate people’s momentary cognitive functioning in their natural environments without the time demand of additional formal cognitive testing. Paradata have been investigated as a means of approximating the cognitive aspects underlying survey responding in traditional (non-EMA) survey studies. For instance, a study examining survey response behaviors at older ages found that the time of survey initiation and time of survey completion were related to mild cognitive impairment [8]. In another study, survey RTs and answer changes were operationalized as indicators of cognitive effort [9]. However, to date, very few studies have investigated the potential use of paradata in EMA surveys as indicators of cognitive performance [10]. One study found a moderate association between the time to complete EMA surveys and processing speed [10] but did not examine the effect of the types of EMA items or the degree of within-person (WP) reliability of EMA RTs.

### This Study

The purpose of this study was to investigate whether the RTs to noncognitive EMA questions (eg, mood, stress, and activity done) can serve as approximate indicators of person-level differences and momentary WP fluctuations in processing speed. Aside from individuals’ average processing speed (person-level differences), momentary fluctuations in processing speed may also be useful to assess via EMA survey paradata. For instance, intraindividual cognitive variability increases with age, even among those who remain cognitively healthy [17], and variability is a risk factor for mild cognitive impairment and Alzheimer disease and related dementias [18-22], even after adjusting for average performance [18]. In addition, WP variability in processing speed, as measured by formal cognitive tests, has been shown to be affected by momentary factors, including caffeine consumption [23], social context [24], and fatigue [14]. We acknowledge that RTs to survey items have been proposed to contain different types of information, including the level of cognitive effort invested [25]; processing speed [26]; and, for self-reports of current mood, the level of emotional clarity [27]. Therefore, survey item RTs likely reflect a combination of several factors. Thus, we did not expect a complete overlap between RTs and the results of mobile cognitive testing but did expect a substantial association.

We capitalized on preexisting data from an EMA study in which adults with T1D completed 2 weeks of EMA surveys together with smartphone-based mobile processing speed and sustained attention tests [16]. Cognitive tests provided validated processing speed and attention measures against which we compared the EMA survey RTs. The makeup of the sample, adults with T1D, allowed for analyses in a sample for which processing speed may be especially relevant. In individuals with T1D, previous studies have found relationships between blood glucose metrics and cognitive performance, including processing speed [11,28,29].

As the primary test of convergent validity, we examined the associations between the RTs to different subsets of EMA items and the scores of mobile processing speed tests. We hypothesized that if EMA survey RTs captured processing speed, slower RTs would be associated with worse performance on the formal processing speed test, both at the between-person (BP) and WP levels.

As secondary tests of convergent validity, we examined the associations between the RTs to different subsets of EMA items and depression symptoms, age, fatigue, and a diurnal cycle. In a review, individuals with major depression were found to have lower processing speed than controls [30], so we expected greater depression symptoms to be associated with slower mean EMA survey RTs. Aging has often been linked to decreased processing speed through various neurobiological pathways [31,32], so greater age was hypothesized to be associated with slower mean EMA survey RTs. We hypothesized that fatigue would have associations with EMA survey RTs at both the BP and WP levels. At the BP level, chronic fatigue syndrome has been associated with slower overall processing speed [33]. At the WP level, processing speed was slower among breast cancer survivors reporting higher than usual fatigue [14]. Finally, cognitive abilities have been found to be at the lowest level during early morning and nighttime, increasing throughout the day until evening [34]. EMA survey RTs were expected to follow a similar diurnal pattern.

To assess divergent validity, we tested the association between EMA item RTs and sustained attention ability. We anticipated that if EMA RTs are indicators of processing speed, they would have stronger associations with processing speed than with sustained attention ability. Although both processing speed and sustained attention ability are fundamental cognitive skills, they are distinct aspects of cognitive performance that are measured using different tests [5,35]. For instance, sustained attention tests are often scored for accuracy, whereas processing speed tests are scored for speed [36].

## Methods

### Study Design

The goal of the EMA study from which data were analyzed was to investigate the relationships among momentary emotion, function, and glucose, the full methodology of which has been outlined previously [16]. Participants were recruited from 3 clinical sites, and the inclusion criteria were as follows: age of >18 years, familiarity with using a smartphone, and sufficient visual acuity, cognitive ability, and manual dexterity to complete study tasks, such as processing speed tests [16]. Consent to participate was provided on the web through the REDCap (Research Electronic Data Capture; Vanderbilt University) e-consent framework [37]. Study procedures included the completion of baseline surveys; 2 weeks of phone-based EMA and cognitive testing with 5 to 6 assessments per day, wearing a continuous glucose monitor and accelerometer during the EMA period; and follow-up surveys.

### Ethics Approval

The data collection procedures were approved by the University of Southern California institutional review board (proposal #HS-18-01014).

### Measures

#### EMA Item RTs

RTs to the 22 noncognitive EMA survey items listed in Table 1 were examined as potential processing speed indicators. All items were derived from validated measures or used in prior EMA research [16]. The items were presented one at a time on study phone screens via the mobile EMA app [38]. The participants were not informed that their EMA survey RTs were being measured. Whether participants should be informed of the collection of paradata continues to be debated [39]. RTs were recorded in seconds (to three decimal places) for each item. Values of <0.2 seconds or >30 seconds were considered missing in analyses (5979/461,896, 1.29% of observations) because prior literature suggested that ultrafast EMA RTs are likely indicative of careless responding [40] and because RTs of >30 seconds were deemed outliers that may have been caused by disruptions in completing the survey. Log transformation was applied to the RTs to create more normal distributions.

**Table 1:** Ecological momentary assessment items from which response times were analyzed.

Item type and question or questions				Response option or options
**16 slider scale items**				
	**Positive affect**			0 (not at all) to 100 (extremely)
		Right now, how content do you feel?		
		Right now, how happy do you feel?		
		Right now, how excited do you feel?		
		Right now, how enthusiastic do you feel?		
	**Negative affect**			0 (not at all) to 100 (extremely)
		Right now, how disappointed do you feel?		
		Right now, how sad do you feel?		
		Right now, how upset do you feel?		
		Right now, how anxious do you feel?		
	**Activity engagement (with reference to the activity the participant reporting doing right before the survey)**			
		How well were you able to do this activity?	0 (unable) to 100 (extremely well)	
		How satisfied are you with the way you did this activity?	0 (not satisfied) to 100 (extremely satisfied)	
		How important is this activity to you?	0 (not important) to 100 (extremely important)	
	**Stress**			
		How stressed are you right now?	0 (not at all stressed) to 100 (extremely stressed)	
		How stressed do you feel about your diabetes or diabetes management right now?	0 (not at all stressed) to 100 (extremely stressed)	
		Right now, how tense do you feel?	0 (not at all) to 100 (extremely)	
	**Fatigue**			
		At this moment, how tired do you feel?	0 (not at all) to 100 (extremely)	
	**Pain**			
		At this moment, how much bodily pain do you have?	0 (none) to 100 (extreme pain)	
**3 multiple-choice items**				
	**Activity done**			
		What were you doing right before starting this survey?	10 choices (eg, work and relaxing)	
	**Where activity was done**			
		Where were you when doing this activity?	5 choices (eg, home)	
	**Subjective blood sugar level**			
		How does your blood sugar feel right now?	Likert 0-4: very low, low, just right, high, and very high	
**3 checkbox items**				
	**With whom**			
		Who were you with when doing this activity?	8 choices (eg, alone and friend)	
	**Diabetes intrusiveness**			
		Did your diabetes get in the way of doing this activity?	4 choices (eg, no and yes because of my devices)	
	**Eat or drink**			
		Did you eat or drink in the last 3 hours?	Ate, drank, and neither	

Our primary set of noncognitive EMA survey RTs was the RTs to the 16 slider items, but other noncognitive EMA survey RT combinations were also examined. To compare the reliability and validity of EMA RTs across types of EMA items, we classified the items by response option type and further by item content domains, where possible, as presented in Table 1. We note that the EMA items were chosen to serve the goal from the overarching study of investigating the relationships between various measures and blood glucose metrics and were not deliberately crafted to ensure a sufficient range of item characteristics. The 16 slider items were designated as primary because they comprised the largest group of items with the same type of response options and thus seemed most likely to have the highest reliability among the EMA item groups. As there were relatively few multiple-choice and checkbox items, we did not create subgroups by item content for the items with these response options. Grouping was applied to the extent feasible to allow for the investigation of the impact of item characteristics on the validity and reliability of EMA RTs as indicators of processing speed. Given the prior findings that the content of survey items and the type of response options may affect RTs [41,42], we carefully examined the extent to which the association between EMA survey RTs and processing speed differed according to these item characteristics.

#### Processing Speed Test

**Figure 1:**
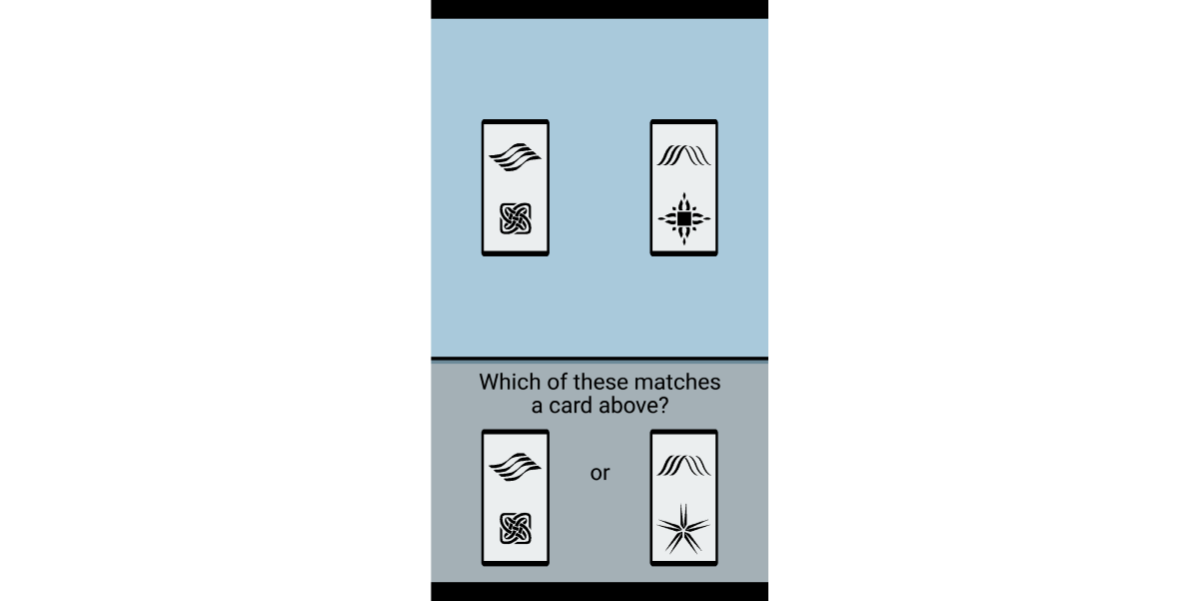
Processing speed test (Symbol Search) on study phone screen, where participants are asked to choose a card on the bottom that matches the card on top as quickly as they can for 20 trials.

At the end of each EMA survey, participants were prompted to complete the Symbol Search task, a phone-based mobile processing speed test [5]. This task has previously demonstrated construct validity and was found to correlate at *r*=0.74 with its standard laboratory version [5]. In this task, participants were presented with 2 cards at the top and bottom of the screen, each with 2 symbols (Figure 1). They were asked to choose the card at the bottom of the screen that matched the card on top as quickly as they could for 20 trials. One Symbol Search session was approximately 45 seconds long. When Symbol Search RTs were <0.2 or >5 seconds (9557/287,543, 3.32% of observations) or they were part of sessions with <70% accuracy (a cutoff for inattentive responding; 2956/287,543, 1.03% of observations) [43], they were considered missing (12,513/287,543, 4.35% of observations).

#### Other Measures

The measures of fatigue, depression, age, and the time of day were also used for validity testing. The momentary fatigue item (ie, “At this moment, how tired do you feel?”) was similar to that used in a previous EMA study [14]. Depression was measured using the Patient Health Questionnaire-8 (PHQ-8) [44] at baseline, along with age. The time of day when each ambulatory assessment was completed was automatically recorded using the EMA app. Sustained attention ability was assessed using an ambulatory cognitive assessment, the Go-No Go task [35], which was administered immediately before the Symbol Search task. In the Go-No Go task, the participants were shown a series of 75 images of a mountain or city, presented one at a time for 800 ms. They were asked to tap an indicated button when seeing an image of a city but to abstain from tapping when presented with an image of a mountain. The measure d prime (d’) was computed as the sustained attention ability score, a metric that considers both the number of correct city taps and incorrect mountain taps using a signal detection approach [35].

### Statistical Analyses

#### Analysis Strategy Overview

Multilevel modeling was used to evaluate (1) the WP and BP reliability of EMA RTs, (2) WP and BP correlations of EMA RTs with the Symbol Search task (primary convergent validity test), and (3) WP and BP associations of EMA RTs with other constructs (secondary convergent validity and divergent validity tests). Analyses of reliability and convergent validity were conducted in a parallel fashion for EMA RTs and for the Symbol Search task. Thus, the results of the reliability and validity testing of EMA RTs could be directly compared with the findings from the Symbol Search analyses. For instance, the magnitude of the correlation between EMA RTs and fatigue can be compared with the size of the association between the Symbol Search task and fatigue.

To examine the extent to which the characteristics of EMA items may impact the reliability and validity of EMA RTs, analyses were conducted using the RTs to all 22 EMA items, as well as using the RTs to subgroups of EMA items and the RTs to individual items. The groupings of item RTs were as presented in Table 1: 16 slider scale items, 4 positive affect slider items, 4 negative affect slider items, 3 activity engagement slider items, 3 stress slider items, 3 multiple-choice items, and 3 checkbox items. The 16 slider items consisted of the slider item subgroups and slider items addressing fatigue and pain in combination. Single-item EMA RTs from each of the 22 EMA items were also included in the analyses as the lower benchmarks of the reliability and validity of EMA RTs provided by this minimal information source and to examine the extent to which the various individual item RTs provided similar or markedly different information when used as an indicator of processing speed.

#### Reliability

Both the BP and WP reliabilities of EMA RTs and the Symbol Search task were estimated. BP reliability describes the consistency of a person’s mean value across all measurement occasions of a given measure (ie, RTs) [45]. It can be calculated as BP reliability = Var(BP)/(Var(BP) + Var(WP)/n) [46], where Var(BP) is the BP variance in the average of scores across measurement occasions, Var(WP) is the variance of scores across measurement occasions within a person, and n is the number of measurement occasions. The equation implies that greater WP variation in EMA RTs decreases the consistency of the average of RTs across all measurement points and that a greater number of measurements (eg, more EMA surveys) increases reliability. An average of 70 surveys were completed over the 2 weeks of the study, and we estimated the BP reliabilities for increasing numbers of measurement occasions (from 2 to 70). This allowed the examination of how BP reliability increased as a function of the number of ambulatory assessments. The variance components Var(BP) and Var(WP) were estimated using a 2-level multilevel model, in which measurement occasions were nested in participants. BP intraclass correlation coefficients (ICCs) were also computed for each measure, which represent BP reliabilities associated with a single measurement occasion (ie, n=1).

To estimate the WP reliabilities of the measures, we capitalized on the fact that the RTs from multiple EMA items (and from multiple Symbol Search trials) were available at each measurement occasion (ie, at each EMA prompt). WP reliability is the consistency of the mean RT across EMA items within a single measurement occasion. The formula is WP reliability = Var(WP_occasion_)/(Var(WP_occasion_) + Var(WP_trial_)/i) [47], where Var(WP_occasion_) is the variance within a person across different measurement occasions, Var(WP_trial_) is the variance of RTs across the EMA items administered within a given measurement occasion, and *i* is the number of EMA items. The variance components Var(WP_occasion_) and Var(WP_trial_) [48] were estimated using 3-level multilevel models (EMA items or trials nested in measurement occasions nested in people).

#### Validity

As the primary convergent validity test, the WP and BP correlations of EMA RTs with the Symbol Search task were examined using bivariate multilevel models, in which both measures were entered as bivariate (ie, correlated) dependent variables. Specifically, 3-level models (items nested in measurement occasions nested in people) were specified, whereby the WP correlation was estimated at level 2 and the BP correlation was estimated at level 3. This had the advantage that the correlations at both levels were adjusted for the unreliability due to variance in RTs within measurement occasions (estimated at level 1). For exploratory purposes, 2-level models were also examined to allow for comparison with the results from the 3-level models. In these 2-level models, rather than estimating the variance in RTs within measurement occasions at level 1, we used the observed (manifest) average of RTs.

Secondary convergent validity and divergent validity tests were conducted similarly with 3-level multilevel models. As fatigue ratings and Go-No Go (sustained attention ability) varied both within and between individuals, we estimated both between-individual and within-individual correlations of fatigue and Go-No Go with EMA RTs (and, for comparison, with the Symbol Search task, examined in separate models). For the BP variables age and depression, we estimated only BP correlations with EMA RTs (and with the Symbol Search task, in separate models). The diurnal cycle of EMA RTs was also examined to test whether the pattern was consistent with previous research on the diurnal cycle of cognitive performance. A multilevel cosinor model [49] was used, in which EMA RTs for a measurement occasion were regressed on the sine and cosine of the hour (0-24 hours) during which the survey was conducted. A 3-level multilevel model (EMA items nested in measurement occasions nested in individuals) was used again, in which EMA RTs were regressed on the sine and cosine of the time of day at level 2 to estimate WP changes in EMA RTs by the time of day. For comparison, a cosinor model was also tested for the Symbol Search task. All reliability and validity analyses were conducted in M*plus* (version 8.8; Muthén & Muthén) [50] with the R package *MplusAutomation* [48] in the statistical software R (R Foundation for Statistical Computing) [51].

## Results

### Sample Characteristics

The analyses were conducted on data from 198 participants (Table 2). A total of ≥4 EMA prompts (together with Symbol Search assessments) were completed on 81.9% (2321/2834) of the data collection days pooled across all participants. The median EMA completion rate over the 2-week study period was 92%. The mean score on the PHQ-8 was 5.44 (SD 4.30), with scores of >9 indicating moderate or more severe depressive symptoms. Overall, 15.7% (31/198) of the participants had PHQ-8 scores of >9. In terms of fatigue, the mean level reported in EMA was 42.70 (SD 18.60), with ratings given on a scale ranging from 0 to 100.

**Table 2:** Demographic and health characteristics (n=198).

Characteristic			Values
Age (years), mean (SD; range)			39.8 (14.4; 18-75)
**Gender, n (%)**			
	Men	89 (44.9)	
	Women	109 (55.1)	
**Ethnicity, n (%)**			
	White	57 (28.8)	
	Latino	81 (40.9)	
	African American	29 (14.6)	
	Multiethnic	14 (7.1)	
	Asian	7 (3.5)	
	Other	6 (3)	
	Not reported	4 (2)	
**Preferred language, n (%)**			
	English	177 (89.4)	
	Spanish	21 (10.6)	
**Employment status, n (%)**			
	Full time	70 (35.4)	
	Part time	23 (11.6)	
	Full-time homemaker	10 (5.1)	
	Student	18 (9.1)	
	Unemployed	27 (13.6)	
	Retired	15 (7.6)	
	Disabled	23 (11.6)	
	Other	8 (4)	
	Not reported	4 (2)	
**Education, n (%)**			
	High school graduate or less	50 (25.3)	
	Some college	68 (34.3)	
	Bachelor’s degree	55 (27.8)	
	Graduate degree	22 (11.1)	
	Not provided	3 (1.5)	
**Annual household income (US $), n (%)**			
	<25,000	48 (24.2)	
	25,000-49,999	44 (22.2)	
	50,000-74,999	15 (7.6)	
	≥75,000	40 (20.2)	
	Not provided	51 (25.8)	
Average blood glucose over at least 10 days of CGM^a^ data (mg/dL; n=154), mean (SD; range)			183.6 (55.0; 98.5-419.8)
Time since T1D^b^ diagnosis (years; n=195), mean (SD; range)			20.9 (12.6; 1-57)
**Insulin delivery system, n (%)**			
	AID^c^	45 (22.7)	
	Non-AID CGM	70 (35.4)	
	No CGM	83 (41.9)	

^a^CGM: continuous glucose monitor.

^b^T1D: type 1 diabetes.

^c^AID: automated insulin delivery.

### Reliability

The reliabilities of the Symbol Search task and EMA RTs are listed in Table 3. The Symbol Search task had a BP ICC of 0.71, suggesting acceptable BP reliability from 1 assessment. With 3 measurement occasions, the BP reliability for the Symbol Search task increased to 0.88. The WP reliability of the Symbol Search task was 0.76, indicating acceptable consistency (>0.7) [52] of the RTs within a single Symbol Search measurement occasion.

**Table 3:** Reliability of the response times to the Symbol Search task and different sets of ecological momentary assessment (EMA) items.

	20 SS^a^ trials	16 slider items	4 positive affect items	4 negative affect items	3 activity engagement items	3 stress items	3 MC^b^ items, 5-10 choices	3 checkbox items, 3-8 boxes	Slider, MC, and check box items
ICC^c^	0.71	0.56	0.44	0.43	0.42	0.44	0.31	0.39	0.56
Between-person reliability of the mean of 3 EMA surveys	0.88	0.79	0.70	0.70	0.69	0.70	0.57	0.66	0.79
Between-person reliability of the mean of 70 EMA surveys	0.99	0.99	0.98	0.98	0.98	0.98	0.97	0.98	0.99
Within-person reliability	0.76	0.82	0.58	0.59	0.60	0.49	0.08	0.22	0.80

^a^SS: Symbol Search (higher values indicate worse processing speed).

^b^MC: multiple-choice.

^c^ICC: intraclass correlation coefficient.

EMA RTs from the 16 slider items had a BP ICC of 0.56, which was lower than the BP Symbol Search ICC. With 3 measurement occasions, the BP reliability of EMA RTs from the 16 slider items increased to 0.79. The WP reliability of EMA RTs from the 16 slider items was 0.82, which was slightly higher than that of the Symbol Search task. The BP and WP reliability values of EMA RTs from all 22 EMA items were nearly identical to those from the 16 slider items.

In terms of RTs from other sets of EMA items comprising 3 to 4 questions, BP ICCs ranged from 0.31 for the 3 multiple-choice items to 0.44 for the 4 positive affect slider items. With 3 measurement occasions, only the BP reliability of the RTs for the 3 multiple-choice items was notably <0.70, with a value of 0.57. WP reliability for the EMA RTs of the slider item subgroups ranged from 0.49 to 0.60. RTs for the 3 multiple-choice and 3 checkbox items had much lower WP reliabilities, with values of 0.08 and 0.22, respectively.

**Figure 2:**
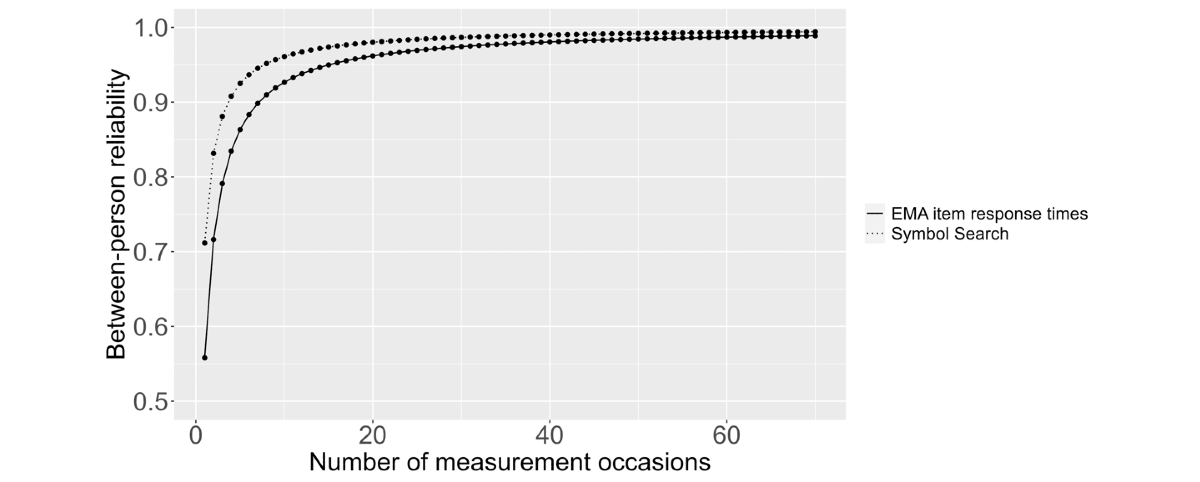
Between-person reliability of the Symbol Search task and ecological momentary assessment (EMA) response times to 16 slider items by the number of measurement occasions.

Figure 2 depicts how the BP reliability for both the Symbol Search task and EMA RTs to 16 slider items varies as a function of the number of measurement occasions. For the Symbol Search task, just 1 measurement occasion is sufficient for a BP reliability of at least 0.70. RTs for 16 slider items crossed the threshold of 0.70 BP reliability upon the completion of 2 EMA surveys.

**Figure 3:**
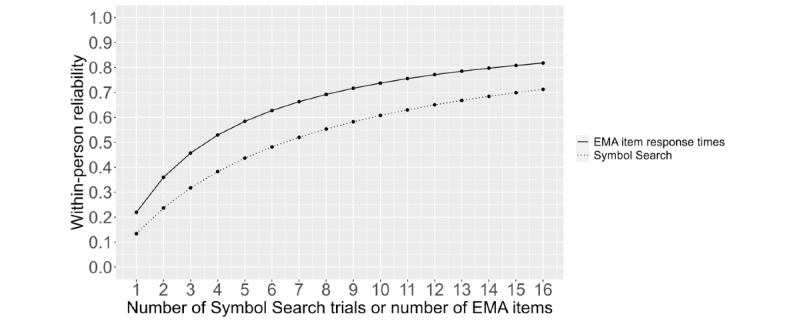
Within-person reliability of the Symbol Search task and ecological momentary assessment (EMA) response times to 16 slider items by the number of trials or the number of EMA items completed. Note that each Symbol Search session had 20 trials, but only the reliability of up to 16 trials was plotted in the figure to correspond with the 16 slider items.

Figure 3 shows the WP variability of the Symbol Search task and RTs to 16 EMA slider items as a function of the number of Symbol Search trials or EMA items completed. Increasing the number of EMA RTs to items of the same type (here slider) steadily increased WP reliability, and the rate of increase roughly mirrored that of the Symbol Search task. For the Symbol Search task, 16 trials were required for a WP reliability of at least 0.70. EMA RTs for 16 slider items crossed the threshold of 0.70 WP reliability upon the completion of 9 items.

BP reliabilities for the RTs to single EMA items are presented in Tables S1 and S2 in Multimedia Appendix 1. Note that using RTs from a single item does not allow for the calculation of WP reliability. Overall, the BP reliabilities of single items from a single EMA measurement occasion (ICC) ranged from 0.17 for the multiple-choice activity engagement item to 0.36 for the diabetes stress item. For the average of 3 measurement occasions, the BP reliabilities of the RTs to single EMA items ranged from 0.50 to 0.63. For the average of 7 measurement occasions, BP reliabilities were at least 0.7 for all items except the multiple-choice items.

### Validity

#### Associations Between EMA RTs and the Symbol Search Task

At the BP level, the correlation between the Symbol Search task and EMA RTs was 0.58 when all EMA items were used, and correlations ranged from 0.49 to 0.57 when subsets of EMA items were used to estimate person-level average EMA RTs (Table 4). At the WP level, medium associations were found between the Symbol Search task and EMA RTs for all item sets except the multiple-choice items, with correlations ranging from *r*=0.29 (*P*<.001) to *r*=0.40 (*P*<.001); multiple-choice items had a larger correlation with the Symbol Search task (*r*=0.58, *P*<.001; refer to the “within-person correlations” category in Table 4).

**Table 4:** Between-person (person-level) correlations between response times from different items (columns) and other variables (rows) as calculated from 3 level models.

				20 SS^a^ trials		16 slider items		4 PA^b^ slider items		4 NA^c^ slider items		3 activity slider items		3 Stress slider items		3 MC^d^ items		3 check items		22 slider, MC, check
**Between-person correlations**																				
	**SS**																			
		*r*	1.00		0.55		0.54		0.55		0.49		0.52		0.57		0.55		0.58	
		*P* value	<.001		<.001		<.001		<.001		<.001		<.001		<.001		<.001		<.001	
	**Fatigue**																			
		*r*	−0.07		−0.03		−0.10		0.00		−0.03		−0.01		0.00		0.00		−0.03	
		*P* value	.18		.28		.08		.47		.37		.46		.49		.50		.37	
	**Depression**																			
		*r*	0.06		0.05		0.02		0.05		0.06		0.03		0.10		0.07		0.05	
		*P* value	.20		.26		.38		.23		.23		.32		.08		.17		.29	
	**Age**																			
		*r*	0.42		0.52		0.49		0.53		0.47		0.54		0.52		0.54		0.54	
		*P* value	<.001		<.001		<.001		<.001		<.001		<.001		<.001		<.001		<.001	
	**GNG^e^**																			
		*r*	−0.15		0.17		0.16		0.1		0.11		0.2		−0.08		−0.03		0.1	
		*P* value	<.001		.02		<.001		.08		.07		<.001		.16		.33		.04	
**Within-person correlations**																				
	**SS**																			
		*r*	1.00		0.35		0.30		0.29		0.35		0.35		0.58		0.40		0.37	
		*P* value	<.001		<.001		<.001		<.001		<.001		<.001		<.001		<.001		<.001	
	**Fatigue**																			
		*r*	0.14		0.08		0.06		0.08		0.05		0.06		0.05		0.05		0.07	
		*P* value	<.001		<.001		<.001		<.001		<.001		<.001		.11		<.001		<.001	
	**GNG**																			
		*r*	−0.04		0		−0.01		−0.03		0.01		0.02		0.02		−0.03		0	
		*P* value	<.001		.45		.14		.02		.18		.08		.29		.06		.41	

^a^SS: Symbol Search (higher values indicate worse processing speed).

^b^PA: positive affect.

^c^NA: negative affect.

^d^MC: multiple-choice.

^e^GNG: Go-No Go (higher values indicate better sustained attention ability).

The WP and BP correlations between EMA RTs from single items and the Symbol Search task are presented in Table S3 in Multimedia Appendix 1. To summarize, BP correlations with the Symbol Search task ranged from 0.43 to 0.58, close in magnitude to the BP correlations with RTs from the larger EMA item sets. The WP correlations ranged from 0.09 to 0.21.

#### Secondary Convergent Validity and Divergent Validity Tests

At the BP level, neither the EMA RTs from different sets of items nor the Symbol Search task were significantly associated with average fatigue (*P*=.18) or depression ratings (*P=.*20), contrary to our hypothesis (Table 4). Older age was significantly positively correlated with worse Symbol Search RTs (*r*=0.42; *P*<.001); and age was similarly correlated with EMA RTs, with magnitudes ranging from 0.47 to 0.54 (*P*<.001). Consistent with our hypothesis, EMA RTs were more highly correlated with the Symbol Search scores (*r* values ranging from 0.49 to 0.58; *P*<.001) than with the Go-No Go scores (*r* values ranging from −0.08 to 0.20; 4 of 8 nonsignificant *P* values of .08, .07, .16, and .33).

At the WP level, the correlations were overall consistent with our hypotheses (Table 4). Worse Symbol Search RTs were significantly associated with greater momentary fatigue levels (*r*=0.14; *P*<.001). Slower RTs for EMA items were similarly associated with greater momentary fatigue, and this relationship was significant for RTs from all sets of EMA items (*r* values ranging from 0.05 to 0.08; *P*<.001), except for those from multiple-choice items (*P*=.11). EMA RTs were again more highly correlated with the Symbol Search scores (*r* values ranging from 0.29 to 0.58; *P*<.001) than with the Go-No Go scores (*r* values ranging from −0.03 to 0.02; 7 of 8 nonsignificant *P* values of .45, .14, .18, .08, .29, .06, and .41).

Tables S4 and S5 in Multimedia Appendix 1 show the BP and WP correlations among the study measures, as calculated from 2-level models instead of the 3-level models presented earlier. The greatest difference is that WP correlations between the Symbol Search task and EMA RTs were somewhat lower in the 2-level models (eg, *r*=0.27 in a 2-level model vs *r*=0.35 in a 3-level model for the 16 EMA slider items). RTs from multiple-choice EMA items showed the biggest difference in WP correlation with the Symbol Search task when comparing 2-level and 3-level models (*r*=0.18 in a 2-level model vs *r*=0.58 in a 3-level model).

**Figure 4:**
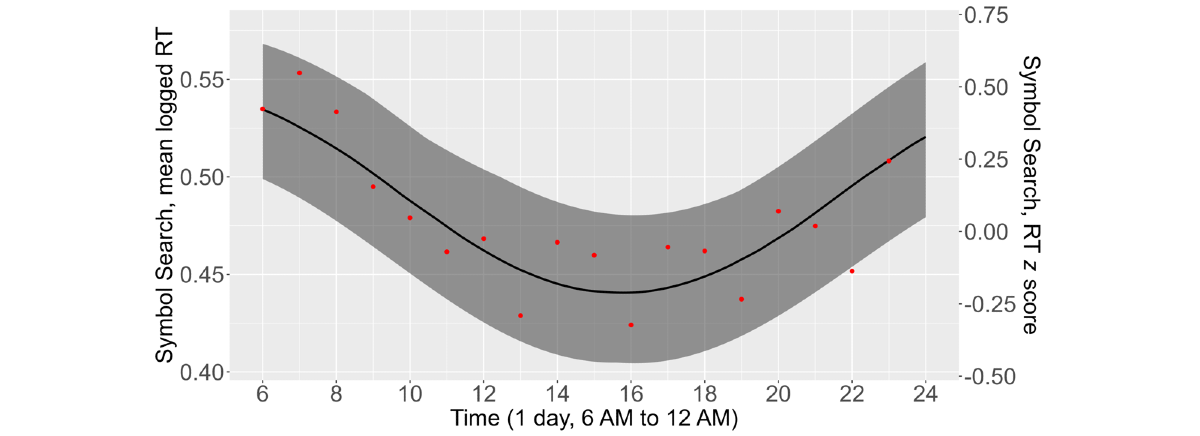
The mean Symbol Search response times (RTs) during typical waking hours (6 AM-12 AM), the period during which most surveys were completed. The black line is the predicted Symbol Search RT from the cosinor model, the band is the 95% CI of the predicted RTs, and the red dots are the observed averages of the Symbol Search RTs.

**Figure 5:**
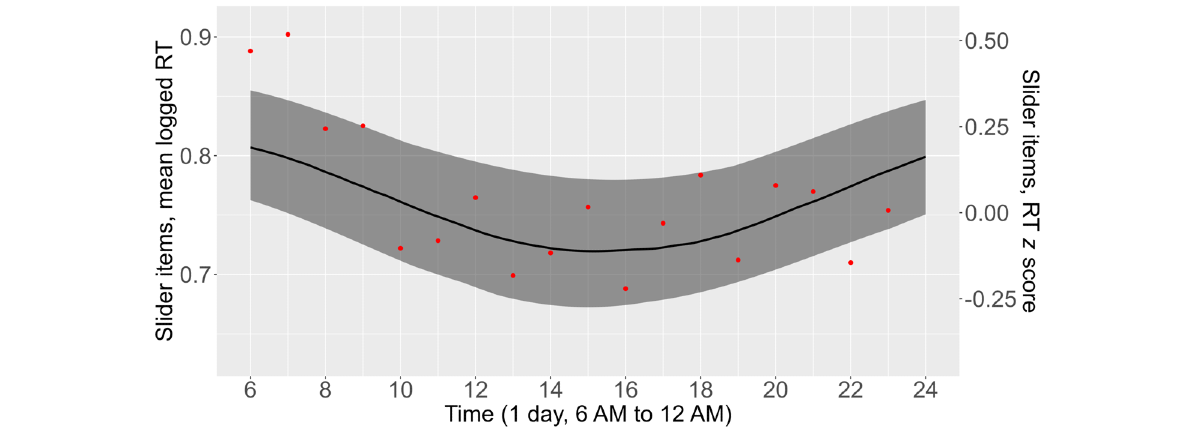
The mean response times (RTs) to 16 slider scale items during typical waking hours (6 AM-12 AM), the period during which most surveys were completed. The black line is the slider scale RT from the cosinor model, the band is the 95% CI of the predicted RTs, and the red dots are the observed averages of the slider scale RTs.

The diurnal cycle of EMA RTs was examined and compared with that of the Symbol Search RTs. As shown in Figure 4, the average Symbol Search RTs were lowest around 3 PM to 4 PM and highest in the morning and evening. The standardized amplitude of the diurnal cycle was 0.34 *z* scores (SE 0.03; *P*<.001), which translated to RT fluctuations of 0.34 × 2 = 0.68 SDs within a day, corresponding to a medium to large effect size [53]. EMA RTs had a similar but less pronounced diurnal cycle (Figure 5). RTs to the 16 slider EMA items were, on average, slowest during the early morning and evening and fastest from 2 PM to 4 PM. The standardized amplitude of the diurnal cycle was 0.16 (SE 0.02; *P*<.001), meaning that RTs fluctuated by approximately 0.16 × 2 = 0.32 SDs (*z* scores) within a day, corresponding to a small effect size [53]. Diurnal plots for the other EMA item sets demonstrated similar trends (not shown here).

## Discussion

### Overview

Overall, our results suggest that EMA RTs can serve as approximate indicators of both momentary and average processing speeds. The EMA RTs for the items analyzed in this study were better indicators of average, as compared with momentary, processing speed. A formal processing speed test (Symbol Search) and EMA RTs had correlations of approximately 0.5 at the BP level and 0.3 at the WP level. These correlation sizes may be acceptable in research contexts in which investigators do not have the resources for administering formal cognitive testing. Correlations of these magnitudes may be sufficient to detect strong associations with processing speed, although weaker associations may be missed. The findings of the reliability and validity tests are described in greater detail in the subsequent sections.

### Reliability

Overall, EMA RTs showed acceptable reliability under conditions (ie, number of items and measurement occasions) typical of many EMA studies. Furthermore, BP reliability for EMA RTs were similar to that for Symbol Search RTs, and WP reliability for RTs to the 16 EMA slider items slightly exceeded that for the Symbol Search task.

The BP reliability of EMA RTs from various item sets differed largely as a function of the number of EMA measurement occasions. With just 3 EMA measurement occasions, BP reliability was acceptable (approximately 0.70), except for RTs from the 3 multiple-choice and 3 checkbox items. The lower BP reliability in these item sets may have been due to their greater heterogeneity (eg, different item content and number of response options), leading to greater variability (ie, more error variance) in mean RTs. For single items, except for multiple-choice questions, the average RTs had a reliability of at least 0.70 with 7 EMA measurement occasions. This suggests that with a relatively small number of EMA measurement occasions, even RTs from single EMA items will likely have acceptable BP reliability. For 16 slider items, the completion of just 2 EMA surveys was sufficient to cross the threshold of 0.70 BP reliability, likely because it was a larger set of items that shared the same type of response options. With more than 16 items sharing similar response options, it would perhaps be possible to obtain a reliable assessment of EMA survey RTs from just 1 measurement occasion.

In terms of the WP reliability of EMA RTs (consistency of RTs within a single EMA measurement occasion), the number of EMA questions and the type of response options appeared to be major contributing factors. The RTs to the 16 slider items had a WP reliability of 0.82, considerably higher than the WP reliability of the RTs to the item sets with only 3 or 4 items. These smaller item sets had reliability values between 0.08 and 0.60, limiting their precision for capturing WP changes in processing speed with 2-level (and not 3-level) modeling. Of the item sets with 3 or 4 items, the more homogenous sets (slider items by topic) had much greater WP reliability than the heterogeneous sets (multiple-choice and checkbox questions with differing numbers of response options). WP reliability improved after combining RTs from various slider items, suggesting that item content was not as important to reliability as the response option type. When considering RTs from the heterogeneous response option item sets, in addition to the 16 slider items, the WP reliability was similar to that found for the 16 slider items alone. Thus, for the WP reliability of EMA RTs, considering RTs from more items may not always be beneficial, specifically when the additional items have different response options.

### Validity

Although some findings were contrary to our hypotheses (ie, no relationship between EMA RTs and depression or fatigue), the results appeared overall supportive of the validity of EMA item RTs as an approximate measure of processing speed. In our primary convergent validity test at the BP level, EMA RTs had moderate to large correlations with the Symbol Search task, a magnitude expected if EMA RTs were indicators of processing speed. In terms of secondary convergent validity tests at the BP level, observed relationships with EMA RTs were sometimes contrary to our hypotheses but were typically very similar to the associations seen with the Symbol Search task. At the BP level, we hypothesized that slower EMA RTs would be associated with greater average fatigue and greater depressive symptoms. Neither of these relationships was confirmed. For fatigue, this may have been because previous research found associations between slower processing speed and chronic fatigue syndrome (more severe than typical fatigue) [33], but the mean level of fatigue reported in the EMA in our sample may have been less severe (mean 42.70, SD 18.60; scale of 0 to 100). In terms of depression, the proportion of people in our sample with any severity of depression (ie, PHQ-8 scores of >9) was 15.7% (31/198), which was greater than the 8.58% (17,040/198,678) found in a previous study on the general population [44]. Associations between depressive symptoms and EMA RTs were trending in the theoretically expected direction (ie, more depressive symptoms were associated with slower processing speed), but our sample may have been underpowered to detect small BP relationships. Symbol Search RTs did not show significant relationships with either fatigue (*P=.*18) or depression (*P=.*20). Significant BP correlations were found between age and both RTs to EMA items (*P*<.001) and the Symbol Search task (*P*<.001). Consistent with our hypothesis, EMA RTs had a greater BP association with the Symbol Search task than with the Go-No Go task. Overall, we interpret the BP correlations as being supportive of the validity of EMA item RTs as approximate measures of processing speed.

At the BP level, higher sustained attention ability was correlated with greater processing speed as measured by the Symbol Search task and was generally weakly associated with EMA RTs. Interestingly, at the BP level, better sustained attention ability was associated with faster Symbol Search RTs but with slower EMA RTs for 4 of the 8 EMA RT item sets. We can only speculate why this might be the case. Perhaps participants with greater sustained attention ability were better able to process information quickly when they were explicitly instructed to respond as fast as possible (in the Symbol Search task), whereas they may have more deliberately read the EMA items and more carefully considered their responses, leading to slower RTs in EMA.

At the WP level, the results were also overall supportive of the validity of EMA item RTs as measures of processing speed. Most importantly, moderate or larger correlations between different sets of EMA RTs and the Symbol Search task were observed. The diurnal cycle of EMA RTs roughly approximated the daily pattern for Symbol Search RTs. Slower EMA RTs were associated with greater fatigue for all item sets, except for multiple-choice questions. Notably, fatigue was associated with processing speed and EMA RTs at the WP level but not at the BP level. It is possible that participants in our sample did at times experience fatigue (within level) but not frequently enough that the average level of fatigue experienced was associated with decrements in processing speed on average (between level). Consistent with our hypothesis, EMA RTs had greater WP associations with the Symbol Search task than with the Go-No Go task.

Although associations between EMA RTs and the Symbol Search task were observed, the relationships were not strong enough to argue that they provided identical measures. From the outset, we did not advocate for RTs of EMA items to serve as a replacement for formal cognitive testing; rather, we sought to examine whether they can serve as rough processing speed indicators when formal tests are not available. The tasks of completing a formal processing speed test and completing EMA items differ in several aspects. For instance, one common formal processing speed test is searching for a figure that matches a given image. In this task, RTs conceptually capture perceptual speed [5], a component of processing speed [31]. RTs in EMA may be more likely to capture decisional speed when faced with a moderately complex task (eg, answering survey items), a conceptually related but different processing speed indicator [31]. As another example, formal processing speed tests often have explicit performance and speed expectations, whereas EMA surveys do not, particularly if participants are not aware that their time to answer questions is being measured. EMA RTs may, therefore, be more affected by distractions because participants may assume that they can attend to distractors and then return to answering EMA questions at their own pace. Given the differences between formal processing speed tests and EMA surveys, their RTs and, by extension, their measures of processing speed were unlikely to correspond exactly with one another. However, because perceptual speed and decision speed both fall under the umbrella of processing speed [31], some associations were expected.

### Validities of EMA RTs With Low Reliabilities

The RTs to single EMA items appeared to lack WP validity, as evidenced by low WP correlations with the Symbol Search task, but they may have some degree of BP validity with sufficient EMA measurement occasions. With an average of 70 EMA surveys completed, the BP association between the RTs to single EMA items and RTs to the Symbol Search task ranged from 0.43 to 0.58. The results of reliability analyses suggested that slightly more than 7 EMAs may result in a BP reliability of at least 0.7, indicating that a relatively small number of EMA instances (eg, 2 days with 4 EMA surveys daily) is sufficient to recover high BP associations with the Symbol Search task.

The RTs to the 3 multiple-choice items notably had the highest WP correlation with the Symbol Search task (*r*=0.58) but also a WP reliability much lower than other item sets. Three-level modeling helped to compensate for this low reliability by removing the errors from item-level RT variance, and the result was a much higher correlation compared with when a 2-level model was used (*r*=0.18). The practical implication may be that EMA RTs with low WP reliability, such as those from a few multiple-choice items differing in content and the number of response options, would not be useful to model with the 2-level approach and requires 3-level modeling. However, with a greater number of parameters specified, 3-level versions of 2-level models require larger sample sizes.

Another implication of the relatively high WP correlation between the RTs to the multiple-choice items and the Symbol Search task may be that multiple-choice question RTs deserve further investigation as potential processing speed indicators, even with the low WP reliability found here. The 3 multiple-choice items asked about activity done before the EMA (from 10 choices), where the activity was done (from 5 choices), and the perceived level of blood glucose (from 4 choices). In a future study, it may be useful to more formally investigate the extent to which item content and the number of response options in multiple-choice questions affect relationships with a formal processing speed test.

### Limitations

The RTs from only a small subset of possible question types were investigated here. For multiple-choice and checkbox items, there were not enough items to investigate the effect of the number of response choices or the topics covered by these response option types. Although the effect of question topic did not appear to exert a large impact on EMA RTs for the slider items in this study, we cannot say whether the content of EMA items influences the reliability or validity of RTs to multiple-choice and checkbox items.

Data from standard laboratory-administered cognitive assessments were not collected. Therefore, we could not examine the convergent validity of individual differences in EMA item RTs using a full-length laboratory assessment of processing speed. Although a previous study found a high correlation between standard laboratory and ambulatory assessments of processing speed [5], whether EMA item RTs are associated with laboratory-based processing speed tests needs to be examined.

The recommended outcome measure for the Symbol Search task, the median reaction time in accurate trials [5], was not used here. This median RT score provides only 1 processing speed measure per Symbol Search session, which does not allow for modeling the Symbol Search scores as a 3-level multilevel model (with items nested in survey sessions nested in people). To allow such modeling, the log-transformed RT for each Symbol Search trial was computed. In preliminary 3-level multilevel model analyses, the mean of the logged RTs of accurate trials (modeled at all levels) correlated with the median RT of accurate trials (modeled at levels 2 and 3), *r*=0.99 (*P*<.001) at the BP level (level 3) and *r*=0.97 (*P*<.001) at the survey session level (level 2). The close correspondence between the 2 appeared to justify the use of mean RTs for accurate trials, instead of the median, to enable the modeling of the Symbol Search task at 3 levels.

This study was conducted with a sample of adults with T1D experiencing various stages of the COVID-19 pandemic, which may limit the generalizability of the results. For instance, study participants were more likely to complete EMA surveys at home during times of stricter social distancing requirements. The completion of EMA surveys at home may have less potential for exposure to environmental distractors, which may have reduced the variability in item RTs. As we only examined EMA RTs as processing speed indicators in adults with T1D, further research may be needed to investigate whether findings can be replicated in other populations. For instance, the causes of processing speed fluctuations are often chronic condition specific. In adults with T1D, acute hypoglycemia has been associated with decreased processing speed [28,29]. In adults with fibromyalgia, greater momentary experiences of pain has been associated with decreased processing speed [54]. The different causes of processing speed fluctuations may also impact the relationship between EMA RTs and scores on formal processing speed tests.

### Conclusions

Overall, EMA RTs appeared to be reliable and valid indicators of average and momentary processing speeds. They were not correlated to the extent that EMA survey RTs can replace formal processing speed tests. Rather, EMA survey RTs may be serviceable as rough processing speed indicators when formal processing speed testing is not feasible and when the magnitude of associations with processing speed is large. The reliability and validity of EMA survey RTs as measures of processing speed differed according to the sets of items from which the RTs were extracted, implying that EMA items can potentially be intentionally crafted to have greater associations with processing speed. For instance, in a future study, factorial analyses or machine learning models could be used to identify the specific combinations of EMA items for which the pattern of RTs is the most predictive of scores from a formal processing speed test. Analysis of RTs from EMA items may be a method of assessing average and momentary processing speeds in people’s natural environments, which does not require participants to complete additional tasks beyond answering EMA survey questions. RTs to noncognitive EMA items may be important to facilitate research on the impacts of processing speed on daily functioning, especially for populations with chronic conditions.

## Appendix

Multimedia Appendix 1 Results of supplementary analyses.

## Abbreviations

BP: between-person
EMA: ecological momentary assessment
ICC: intraclass correlation coefficient
PHQ-8: Patient Health Questionnaire-8
REDCap: Research Electronic Data Capture
RT: response time
T1D: type 1 diabetes
WP: within-person
