# Supplementary material for: Reliability and Validity of Noncognitive Ecological Momentary Assessment Survey Response Times as an Indicator of Cognitive Processing Speed in People’s Natural Environment: Intensive Longitudinal Study
Source: JMIR Mhealth Uhealth. 2023 May 30;11:e45203. doi: 10.2196/45203 (PMC10265432; doi:10.2196/45203)
Supplement: Multimedia Appendix 1 [file mhealth_v11i1e45203_app1.docx]

**Appendix 1**

**Table S1.** Reliabilities of response times to single slider items

|  | Performance | Satisfied | Important | Content | Happy | Enthusiastic | Excited | Anxious |
| --- | --- | --- | --- | --- | --- | --- | --- | --- |
| ICC^a^ | 0.29 | 0.34 | 0.32 | 0.28 | 0.26 | 0.27 | 0.28 | 0.27 |
| Between-person reliability of average of 3 EMA^b^ | 0.55 | 0.61 | 0.59 | 0.53 | 0.51 | 0.52 | 0.53 | 0.53 |
| Average of 7 EMA | 0.74 | 0.78 | 0.77 | 0.73 | 0.71 | 0.72 | 0.73 | 0.72 |
| Average of 70 EMA | 0.97 | 0.97 | 0.97 | 0.96 | 0.96 | 0.96 | 0.96 | 0.96 |

|  | Disappointed | Sad | Upset | Stress | Diabetes Stress | Tense | Fatigue | Pain |
| --- | --- | --- | --- | --- | --- | --- | --- | --- |
| ICC | 0.25 | 0.27 | 0.25 | 0.28 | 0.36 | 0.25 | 0.31 | 0.32 |
| Between-person reliability of average of 3 EMA | 0.5 | 0.52 | 0.5 | 0.54 | 0.63 | 0.5 | 0.57 | 0.59 |
| Average of 7 EMA | 0.7 | 0.72 | 0.7 | 0.73 | 0.8 | 0.7 | 0.76 | 0.77 |
| Average of 70 EMA | 0.96 | 0.96 | 0.96 | 0.96 | 0.98 | 0.96 | 0.97 | 0.97 |

^a^ICC: intraclass correlation coefficient.

^b^EMA: ecological momentary assessment.

**Table S2.** Reliabilities of response times to single multiple- choice and checkbox items

|  | Multiple choice | | | Check all that apply | | |
| --- | --- | --- | --- | --- | --- | --- |
|  | Activity doing (10 choices) | Where doing (5 choices) | Perceived BG level right now (5 choices) | Who with (8 boxes) | Diabetes interfere type (4 boxes) | Ate/drank (3 boxes) |
| ICC^a^ | 0.17 | 0.21 | 0.24 | 0.27 | 0.29 | 0.3 |
| Between-person reliability of average of 3 EMA^b^ | 0.38 | 0.44 | 0.48 | 0.53 | 0.56 | 0.56 |
| Average of 7 EMA | 0.59 | 0.64 | 0.69 | 0.72 | 0.74 | 0.75 |
| Average of 70 EMA | 0.94 | 0.95 | 0.96 | 0.96 | 0.97 | 0.97 |

^a^ICC: intraclass correlation coefficient.

^b^EMA: ecological momentary assessment.

**Table S3.** Between and within-person (EMA level) correlations between response times from individual items (columns) and Symbol Search from a 3 level model (SS trials modeled at level 1)

|  | Perf.^a^ | Satisfied | Important | Content | Happy | Enthus.^b^ | Excited | Anxious | Disapp.^c^ | Sad | Upset |
| --- | --- | --- | --- | --- | --- | --- | --- | --- | --- | --- | --- |
| SS^d^ within-person (EMA^e^ level) | 0.21 (*P*<.001) | 0.21 (*P*<.001) | 0.19 (*P*<.001) | 0.16 (*P*<.001) | 0.16 (*P*<.001) | 0.15 (*P*<.001) | 0.15 (*P*<.001) | 0.14 (*P*<.001) | 0.16 (*P*<.001) | 0.14 (*P*<.001) | 0.14 (*P*<.001) |
| SS between-person | 0.49 (*P*<.001) | 0.46 (*P*<.001) | 0.48 (*P*<.001) | 0.56 (*P*<.001) | 0.49 (*P*<.001) | 0.51 (*P*<.001) | 0.54 (*P*<.001) | 0.53 (*P*<.001) | 0.56 (*P*<.001) | 0.55 (*P*<.001) | 0.58 (*P*<.001) |
|  |  |  |  |  |  |  |  |  |  |  |  |
|  | Stress | Diabetes Stress | Tense | Fatigue | Pain | Activity doing | Where doing | Perceive BG^f^ | Who with | Diabetes interfere | Ate/ drank) |
| SS within-person (EMA level) | 0.15 (*P*<.001) | 0.21 (*P*<.001) | 0.16 (*P*<.001) | 0.2 (*P*<.001) | 0.19 (*P*<.001) | 0.09 (*P*<.001) | 0.13 (*P*<.001) | 0.21 (*P*<.001) | 0.11 (*P*<.001) | 0.16 (*P*<.001) | 0.13 (*P*<.001) |
| SS between-person | 0.52 (*P*<.001) | 0.48 (*P*<.001) | 0.54 (*P*<.001) | 0.54 (*P*<.001) | 0.51 (*P*<.001) | 0.49 (*P*<.001) | 0.51 (*P*<.001) | 0.5 (*P*<.001) | 0.45 (*P*<.001) | 0.43 (*P*<.001) | 0.54 (*P*<.001) |

^a^Perf.: performance.

^b^Enthus.: enthusiastic.

^c^Disapp.: disappointed.

^d^SS: Symbol Search.

^e^EMA: ecological momentary assessment.

^f^BG: blood glucose.

**Table S4.** Between-person correlations between response times from different items (columns) and other variables (rows) as calculated from 2 level models

|  | 20 SS^a^ trials | 16 slider items | 4 PA^b^ slider items | 4 NA^c^ slider items | 3 Act^d^ slider items | 3 Stress slider items | 3 MC^e^ items | 3 Check items | 22 Slider, MC, check |
| --- | --- | --- | --- | --- | --- | --- | --- | --- | --- |
| SS | 1 | 0.56 (*P*<.001) | 0.54 (*P*<.001) | 0.57 (*P*<.001) | 0.5 (*P*<.001) | 0.54 (*P*<.001) | 0.58 (*P*<.001) | 0.55 (*P*<.001) | 0.58 (*P*<.001) |
| Fatigue | -0.08 (*P=.*14) | -0.04 (*P=.*29) | -0.07 (*P=.*15) | -0.01 (*P=.*47) | -0.03 (*P=.*35) | 0 (*P=.*47) | 0.01 (*P=.*43) | 0.01 (*P=.*46) | -0.03 (*P=.*33) |
| Depression | 0.03 (*P=.*27) | 0.05 (*P=.*29) | 0 (*P=.*47) | 0.05 (*P=.*23) | 0.06 (*P=.*18) | 0.04 (*P=.*32) | 0.09 (*P=.*11) | 0.07 (*P=.*11) | 0.04 (*P=.*29) |
| Age | 0.42*** (*P*<.001) | 0.52 (*P*<.001) | 0.48 (*P*<.001) | 0.52 (*P*<.001) | 0.47 (*P*<.001) | 0.54 (*P*<.001) | 0.54 (*P*<.001) | 0.54 (*P*<.001) | 0.56 (*P*<.001) |
| GNG^f^ | -0.12 (*P*=.06) | 0.16 (*P*=.01) | 0.19 (*P*=.02) | 0.12 (*P*=.05) | 0.13 (*P*=.05) | 0.18 (*P*=.03) | -0.08 (*P*=.17) | -0.03 (*P*=.38) | 0.13 (*P*=.06) |

*p<.05; **p<.01; ***p<.001

^a^SS: Symbol Search (higher values indicate worse processing speed).

^b^PA: positive affect.

^c^NA: negative affect.

^d^Act: activity.

^e^MC: multiple choice.

^f^GNG: Go-No Go (higher values indicate better sustained attention ability).

**Table S5.** Within-person correlations between response times from different items (columns) and other variables (rows) as calculated from 2 level models

|  | 20 SS^a^ trials | 16 slider items | 4 PA^b^ slider items | 4 NA^c^ slider items | 3 Act^d^ slider items | 3 Stress slider items | 3 MC^e^ items | 3 Check items | 22 Slider, MC, check |
| --- | --- | --- | --- | --- | --- | --- | --- | --- | --- |
| SS | 1 | 0.27 (*P*<.001) | 0.20 (*P*<.001) | 0.19 (*P*<.001) | 0.22 (*P*<.001) | 0.20 (*P*<.001) | 0.18 (*P*<.001) | 0.16 (*P*<.001) | 0.28 (*P*<.001) |
| Fatigue | 0.12 (*P*<.001) | 0.07 (*P*<.001) | 0.05 (*P*<.001) | 0.06 (*P*<.001) | 0.04 (*P*<.001) | 0.04 (*P*<.001) | 0.01 (*P=.*11) | 0.02 (*P=.*01) | 0.06 (*P*<.001) |
| GNG^f^ | -0.03 (*P*<.001) | 0 (*P*=.46) | -0.01 (*P*=.21) | -0.02 (*P*=.045) | 0.01 (*P*=.15) | 0.01 (*P*=.095) | 0  (*P*=.42) | -0.01 (*P*=.14) | 0  (*P*=.4) |

^a^SS: Symbol Search (higher values indicate worse processing speed).

^b^PA: positive affect.

^c^NA: negative affect.

^d^Act: activity.

^e^MC: multiple choice.

^f^GNG: Go-No Go (higher values indicate better sustained attention ability).
